# Membrane Rafts in T Cell Activation: A Spotlight on CD28 Costimulation

**DOI:** 10.3389/fimmu.2017.01467

**Published:** 2017-11-03

**Authors:** Sara Zumerle, Barbara Molon, Antonella Viola

**Affiliations:** ^1^Department of Biomedical Sciences, University of Padova, Padova, Italy; ^2^Venetian Institute of Molecular Medicine (VIMM), Padova, Italy; ^3^Pediatric Research Institute “Citta della Speranza”, Padova, Italy

**Keywords:** T lymphocyte, CD28 costimulation, membrane rafts, immunological synapse, cytoskeleton

## Abstract

Spatiotemporal compartmentalization of signaling pathways and second messengers is pivotal for cell biology and membrane rafts are, therefore, required for several lymphocyte functions. On the other hand, T cells have the specific necessity of tuning signaling amplification depending on the context in which the antigen is presented. In this review, we discuss of membrane rafts in the context of T cell signaling, focusing on CD28-mediated costimulation.

## Membrane Rafts

In the late 1980 s, accumulating evidence set forth the existence of ordered lipid clusters in the cell membrane, named lipid rafts. According to the “lipid raft hypothesis,” forces between cholesterol and sphingolipids facilitate the dynamic assemblies of lipid domains in an unsaturated glycerophospholipid environment (1). Experimental evidence accumulated during the following years, indicated that in real cell membranes transmembrane, signaling, and cytoskeletal proteins play a key role in controlling raft generation and dynamics, and have suggested a new model based on protein–lipid interaction (2, 3). In 2006, at the end of a vibrant meeting, a consensus definition for membrane rafts was achieved and they were defined as “small (10–200 nm), heterogeneous, highly dynamic, sterol- and sphingolipid-enriched domains that compartmentalize cellular processes” (2, 4). Nonetheless, the size, density, molecular composition, and morphology of these domains in cellular membranes have remained controversial for a very long time.

As a matter of fact, for several years from their initial discovery, scientists were skeptical about the physiological existence of lipid platforms at the cell membrane, mainly because it was almost impossible at that time to probe their presence in living cells. A critical issue in this sense was the methodology employed to define a raft-integrated factor that was foremost based on detergent resistance; indeed, a hallmark of a raft component is the recovery in the low-density fraction after cold Triton extraction and sucrose density gradient centrifugation. However, this technique can produce multiple artifacts due to the type and concentration of the detergents, duration of extraction, and temperature (5, 6). Importantly, methyl-β-cyclodextrin (MβCD) that extracts cholesterol from cell membranes was largely exploited to discriminate whether a protein was a raft component and more to evaluate whether a biological process was raft dependent or independent. Even in this case, the use of this drug was matter of debate due to the multiple non-specific effects of MβCD in addition to cholesterol removal, as lateral protein immobilization, plasma membrane depolarization, and Ca^2+^ store depletion (7).

To overcome biochemical hurdles, alternative approaches with high temporal and spatial resolution have been optimized. Subczynski and Kusumi advanced and exploited pulse electron paramagnetic resonance spin labeling methods and single molecule optical imaging to track the dynamic entry and exit of molecules in membrane rafts (8). In addition, over the last decades a plethora of advanced imaging techniques have been applied to identify membrane rafts in live cells as single fluorophore tracking (9) and photonic force microscopy (10), fluorescence resonance energy transfer (11), fluorescence recovery after photobleaching (12), total internal reflection fluorescence (13), and stimulated emission depletion microscopy (14). For technically oriented readers, distinguished reviews in the field have illustrated advantages and limits of the different methodologies that have been exploited to identify and study lipid membrane microdomains (15).

Beyond conceptual and technical criticisms, it was clear that one of the most relevant features of membrane rafts is their intrinsic capacity to selectively include or exclude specific proteins. The partitioning of a single molecule in such liquid-ordered domains depends on different factors, as cell type and activation state, and more importantly it can be regulated by intra- and extracellular signals (8). Given their small size, the number of proteins that can localize in a single raft domain is limited, probably no more than 10–30 proteins. Controversial theories have been proposed on raft composition in terms of proteins and lipids and several studies have documented the coexistence of different membrane rafts with distinctly different proteins and/or lipids within the same cell type (16). Indeed, a simplified but still operating view of membrane rafts is that they can act as highly dynamic platforms at the plasma membrane, where effector enzymes, cofactors, adaptors, and scaffold proteins can trigger and organize multiple signaling cascades. In this sense, the existence of plasma membrane microdomains not only changes the structural organization of the Singer and Nicolson fluid mosaic model but, importantly, it also influences our knowledge on the mechanisms that connect receptor stimulation with the activation of an intracellular signaling process. Indeed, raft domains may control signal transduction in multiple ways. For example, receptor stimulation can rapidly and efficiently activate a signaling cascade thanks to the spatial juxtaposition of different interacting molecules within the same membrane domain. In addition, as for of tyrosine kinases, rafts can offer a protective micro-environment that isolates the activated signaling machinery from non-raft enzymes such as membrane phosphatases that could inhibit the process. By internalizing selective molecules, membrane rafts also play a role in signal termination (17).

Although originally thought as lipid-based free-floating platforms—“rafts,” indeed—it became clear that membrane rafts are tightly connected with the cell cytoskeleton through actin-binding proteins such as ezrin and filamin (3) and, therefore, more similar to “flying kites” than “floating rafts” (2). The cross-talk between membrane rafts and the actin cytoskeleton is very complex: membrane rafts are indeed enriched in actin-binding proteins (18, 19); they contain phosphatidylinositol-4,5-bisphosphate (PIP_2_) (20, 21), which is a key regulator of the actin-polymerization machinery that, on the other hand, regulates raft assembly and dynamics (22, 23).

As for membrane rafts in immunity, over the last decades it has become evident that these microdomains actively modulate immune signaling, contributing to orchestrate innate and acquired immune responses (24). Compartmentalization of signaling molecules represents indeed an essential strategy exploited by immune cells to integrate multiple extracellular cues and trigger and direct complex functions (25). In T lymphocytes, this is primarily achieved by the allocation of selected proteins into well-organized structures, such as the immunological synapse (IS)—during antigen recognition—or the leading and rear edges—during cell migration—and, importantly, by the clustering of key signaling players into membrane rafts.

This review will describe how membrane rafts support T cell activation, focusing in particular on CD28-mediated costimulation.

## Membrane Rafts in T Cell Activation

In order to become fully activated, T cells require a double signal. The first signal is T cell receptor (TCR) ligation by peptide-major histocompatibility complex on antigen-presenting cells (APCs); the second signal derives from the interaction between a coreceptor on the T cell with counter-receptors expressed by APCs. The major coreceptor molecule expressed by T cells is CD28, whose ligands on professional APCs are CD80/B7.1 and CD86/B7.2 (26–28).

The combination of antigen recognition and costimulation results in the formation of a mature IS (29), a dynamic structure whose role is to regulate T cell function by integrating different signals and, thus, determining the fate of an antigenic stimulation. The IS, also defined as supramolecular activation cluster (SMAC), is described as a “bull’s eye structure,” organized in a central region (c-SMAC) and a peripheral region (p-SMAC) (30). Several proteins involved in T cell signaling, such as TCR/CD3, CD28, protein kinase Cθ (PKCθ), lymphocyte specific protein-tyrosine kinase (Lck), and zeta-chain-associated protein kinase 70 (ZAP-70) are located in the c-SMAC, surrounded by a ring of cytoskeletal or adhesion molecules, forming the p-SMAC, which provides structural support to the IS. Antigen recognition occurs in the c-SMAC, while the p-SMAC maintains the architecture of the IS and stabilizes the T cell–APC conjugation. The spatial organization of the IS is intimately correlated to its function and ensures a fine regulation of T cell activation: indeed, the integration of different signals occurring at the level of the IS ensures both the sensitivity and the specificity of T cell responses.

The IS is characterized by a peculiar lipid composition, which differs from the lipid composition of other membrane regions (31). Live-cell imaging experiments have elegantly shown that raft lipid localization is regulated during the IS formation: rafts first enrich in the center of the synapse and then move to the periphery (31, 32).

Importantly, the functional relevance of membrane rafts is supported by the observation that several signaling molecules involved in T cell activation can be found in raft domains. The Src kinase Lck, which is activated immediately after TCR triggering, is acylated on two cysteine residues on its N-terminus, thus being targeted to membrane rafts (33). This is crucial for Lck-mediated tyrosine phosphorylation, as demonstrated by the fact that Lck mutants resistant to acylation do not localize to the plasma membrane and do not fully activate downstream signaling (34). Also the linker for activation of T cells (LAT) localizes to raft domains. Upon TCR engagement, LAT is phosphorylated by ZAP-70 and, consequently, activates several signaling pathways (35, 36). Mutations preventing LAT palmitoylation inhibit LAT phosphorylation by ZAP-70 and hinder TCR signal transduction (37, 38). Also PKCθ is localized to membrane rafts and clusters at the IS in CD3/CD28-stimulated T cells; PKCθ raft translocation depends on Lck and is crucial for downstream NF-κB activation (39). In addition, the activation of phosphoinositide 3-kinase (PI3K)/Akt signaling pathway, which is crucial for T cell activation, has been shown to be tightly connected to the presence of raft nanodomains (40).

Altogether, these results support a model in which lipid rafts act as a docking platform, where the actors involved in T cell activation can organize and integrate downstream signaling events.

In T cells, the coupling of the outer and inner lipid microdomains is mediated by cholesterol. By contrast, the crosslinking of TCR/CD3 triggers the formation of large, stable rafts independently of cholesterol (41). These results are explained by the role of CD28 in the generation of membrane rafts.

## CD28 Costimulation in T Cell Activation

The costimulatory molecule CD28 plays a crucial role in determining T cell sensitivity. Its ligands CD80 and CD86 are highly expressed by pathogen-activated professional APCs, such as mature dendritic cells, macrophages, and activated B cells. As a consequence, CD28 costimulation occurs in the context of infection and/or inflammation, thus representing a possible control mechanism for avoiding excessive T cell responses. Accordingly, CD28 has been indicated as an important regulator of autoimmune diseases (42). Interestingly, genome-wide studies on patients have associated single nucleotide polymorphisms in the CD28 gene to increased risk for autoimmune disease (43). Notably, two recent works appointed CD28 signaling pathway as the direct target of PD-1 based-therapy, thus providing intriguing insights for the improvement of current immune-therapies against cancer and viral infections (44, 45).

CD28 costimulation is indeed fundamental for full T cell activation, as it lowers the stimulation threshold of naïve T cells, in terms of number of triggered TCRs (28), preventing anergy and enhancing cytokine production, such as interleukin-2 (IL-2), and lymphocyte proliferation (46). T lymphocytes from CD28 knockout mice can be activated by increasing antigen doses, showing that the signals delivered by CD28 can be replaced, at least in part, by stronger TCR signaling (27, 47). These results suggest that CD28 functions as a general amplifier of early TCR signaling, thereby determining the sensitivity of the adaptive immune response. However, several studies have highlighted that CD28 regulates T cell activation not only quantitatively but also qualitatively (48).

Numerous works have demonstrated that CD28 costimulation regulates T cell function, by enhancing several signaling pathways and, finally, tuning gene transcription (49, 50). This can be achieved thanks to the presence of highly conserved tyrosine- and proline-rich signaling motifs on CD28 cytosolic tail, which are phosphorylated following CD3/CD28 stimulation and act as docking sites for the recruitment through their SH2 and/or SH3 domains of several downstream factors (51), such as PI3K (52, 53), Tec and Itk protein-tyrosine kinases (54–56), growth factor receptor-bound protein 2 (Grb2) (57), Grb2-related adaptor protein (Gads) (58), Lck (59), PKCθ (60, 61), Filamin A and associated NF-κB inducing kinase (22, 62), the guanine nucleotide exchange factor Vav1, and associated phosphatidylinositol 4,5-biphosphate kinases (PIP5Ks) α and β (23, 63).

Importantly, CD28-mediated costimulation is intrinsically linked to the reorganization of membrane rafts at T lymphocyte plasma membrane.

## CD28 Costimulation and Membrane Rafts

We and others have shown that CD28 coengagement results in membrane rafts clustering at the site of TCR triggering (22, 64–69). Rafts recruitment into the IS requires indeed CD28 signaling and is not observed in T cells stimulated by CD3-specific antibodies or through the TCR only (22, 64, 69). In addition, CD28-induced membrane raft assembly may precede TCR triggering and prepare a signaling microdomain for the upcoming IS (70).

CD28-mediated raft recruitment is a central event for the orchestration of TCR signaling. Indeed, CD28 coengagement by Abs or natural ligands protects the action of TCR-activated kinases from the inhibition mediated by phosphatases. Consequently, tyrosine phosphorylation of TCR signaling targets is stabilized and can be observed for several minutes, while being transient and unstable in the absence of CD28 costimulation (64). Quantitative phosphoproteomic analysis confirmed that CD3/CD28 stimulation enhances the phosphorylation of sites that are usually modified by CD3 stimulation (71).

As mentioned before, several proteins involved in TCR signaling have been found associated with membrane rafts and, at least for some of them, the recruitment to the IS depends on CD28-triggered organization of lipid domains. Lck, for example, accumulates at the IS in a CD28-dependent way and this process requires the CD28 COOH-terminal PxxPP motif and Vav1 (69).

Also PKCθ clusters at the IS upon CD3/CD28 engagement translocating to membrane rafts (39). Its association with CD28 is necessary for the activation of downstream signaling as NF-κB pathway and plays a crucial role for the differentiation of Th2 and Th17 subtypes, but not Th1 (61, 72, 73). The lymphoid cell-specific actin-uncapping protein Rltpr binds to CD28 and is required for the relocation of PKCθ and CARD-containing MAGUK protein 1 (Carma1) at the central region of the IS (74, 75).

The group of Tasken has described a further CD28-dependent mechanism of T cell regulation, involving cyclic adenosine monophosphate (cAMP) and protein kinase A (PKA). It has been known for a long time that TCR ligation induces an increase in cAMP production (76), which in turns results in PKA recruitment to lipid rafts *via* ezrin and leads to the inhibition of T cell function and proliferation (77). However, when the costimulatory signal delivered by CD28 is triggered, the phosphodiesterase enzyme PDE4 is recruited to lipid rafts; PDE4 locally degrades the TCR-induced cAMP pool, thus counteracting cAMP-mediated inhibition of T cell functions (78, 79). The recruitment of PDE4 to lipid rafts depends on CD28 (78) and involves PI3K activity and PIP_3_ production (79).

The dynamic and functional assembly of the IS depends on actin cytoskeleton rearrangements (80). The actin cytoskeleton drives the contact between the T cell and the APC not only mechanically but also directly interacting with IS-associated signaling molecules (81). As stated above, several components of the actin-based cytoskeleton are enriched in lipid rafts purified from T cells, as highlighted by mass-spectrometry-based proteomic analysis (3, 18, 19).

One of the most characterized functions of CD28 is the ability to organize the cytoskeleton. At the immune synapse, the CD3/CD28-activated polymerization of actin is regulated by the activity of the guanine nucleotide-exchange factor Vav1 (82, 83), the small Rho GTPase cell division control protein 42 (Cdc42) (84), the Wiskott–Aldrich Syndrome protein (WASP) (66) and the actin-related protein 2/3 complex (ARP2/3). The ARP2/3 complex interacts with filamins, elongated proteins that crosslink F-actin thus generating and organizing dynamic cytoskeletal networks (85). We demonstrated that, after physiological stimulation, CD28 recruits Filamin A at the IS (22), where Filamin A regulates TCR and CD28 signaling (22, 86). Interestingly, the absence of Filamin A results in decreased membrane raft clustering at the IS along with impaired CD28-dependent costimulation (22). Furthermore, Filamin A seems to contribute to the Vav1-dependent actin-polymerization pathway to regulate raft dynamics by confining them at the IS (3, 22).

Vav1 is a key regulator of cytoskeletal dynamics and T cell responses: indeed, Vav1-deficient cells present impaired membrane raft clustering at the IS, which correlates with defects in cytoskeletal reorganization and T cell activation (67, 87, 88). CD28, *via* the adaptor protein Grb2, mediates the formation of Vav1/SLP-76 complex, which is crucial for initiating downstream signaling (82, 83).

The role of Vav1 in CD28-mediated signaling is crucial: T cells lacking the adaptor molecule Cbl-b display enhanced Vav1 activation and are independent of CD28 for their triggering (89), as well as for membrane raft clustering at the IS (90). In the absence of Cbl-b, the deregulated activation of the Vav1/WASP pathway induces receptor clustering irrespective of CD28 signaling and enhances TCR signaling (90). In agreement, WASP-deficient T cells show defects in membrane raft reorganization in response to CD28 costimulation (66).

Also cofilin has been proposed as a linker between the actin cytoskeleton and T cell costimulation. Indeed, TCR/CD28 engagement, but not TCR stimulation alone, induces cofilin dephosphorylation and its consequent association with actin (91). In T cells, cofilin dephosphorylation can be mediated by the protein phosphatases PP1 and PP2A (92); interestingly, PP2A has been reported to interact with CD28 (93), but the functional significance of this interaction has not been elucidated yet.

Cytoskeletal dynamics are also regulated by PIP_2_, produced by PIP5K enzymes. It has been estimated that half of the PIP_2_ pool is localized in membrane rafts (20) and is important, for example, for the recruitment of WASP (94). Recent studies show that upon CD28 costimulation, PIP5Kβ translocates to the IS where it organizes both the recruitment of filamin A and membrane raft clustering. Moreover, in collaboration with PIP5Kα and Vav1, PIP5Kβ promotes actin polymerization and CD28 signaling in human T cells (23, 63, 95, 96).

## Concluding Remarks

**Figure 1:**
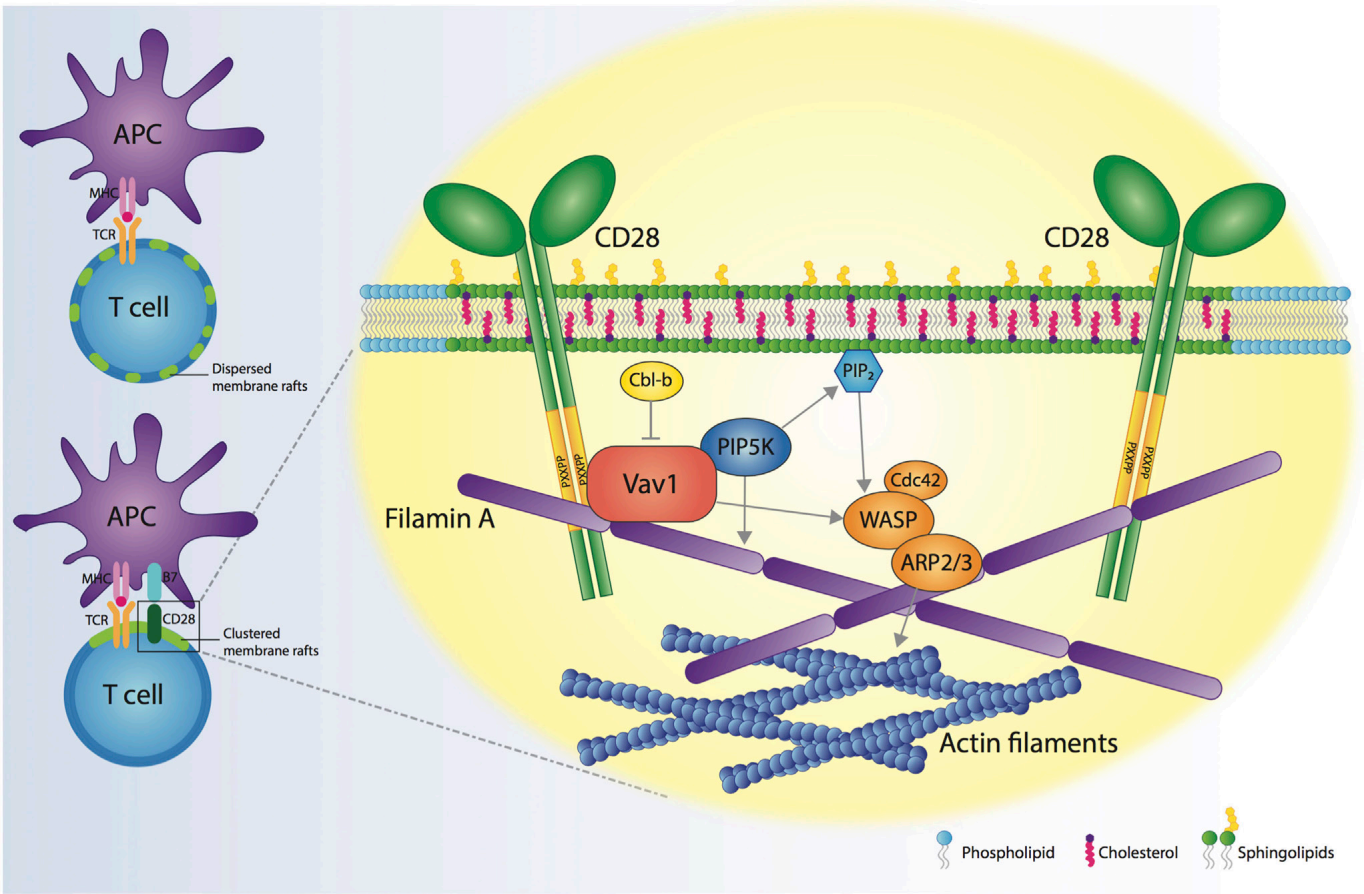
CD28 orchestrates membrane raft trapping at the immunological synapse (IS). The engagement of both the T cell receptor (TCR) and CD28 by the interaction with a professional antigen-presenting cell (APC) results in the formation of a mature IS, characterized by the clustering of membrane rafts. CD28 cytosolic tail binds Filamin A and the nucleotide-exchange factor Vav1, thus regulating the action of cell division control protein 42 (Cdc42), Wiskott–Aldrich Syndrome protein (WASP) and actin-related protein 2/3 complex (ARP2/3) proteins, which are responsible for increased actin polymerization at the IS. CD28, through Vav1, recruits phosphatidylinositol 4,5-biphosphate kinases (PIP5Ks) to the IS and promotes PIP5K-dependent production of phosphatidylinositol-4,5-bisphosphate (PIP_2_). PIP5Ks are pivotal for the recruitment of Filamin A and membrane rafts and sustain actin polymerization. The molecular interactions initiated by CD28 result in the cytoskeletal reorganization associated with membrane raft clustering at the IS.

Since the first demonstration in 1999 for a role of CD28 in controlling membrane rafts assembly at the IS (64), several studies have contributed to identify the molecular mechanisms responsible for the formation of these selective and dynamic signaling microdomains. Today, we know that CD28-dependent recruitment of membrane rafts at the IS depends on specific residues of the CD28 cytoplasmic domain that allow CD28 to recruit Vav1 and initiate signaling pathways involving PIP5Ks and actin-binding proteins (Figure 1). This massive actin reorganization allows recruitment and trapping of membrane rafts in the IS and is required to sustain and amplify TCR-induced signaling when costimulation is provided. Thus, CD28-mediated assembly of membrane rafts represents an extraordinary strategy for spatiotemporal compartmentalization and context-specific amplification of signal transduction.

## Author Contributions

SZ, BM, and AV conceived and wrote the manuscript and realized the figure.

## Conflict of Interest Statement

The authors declare that the research was conducted in the absence of any commercial or financial relationships that could be construed as a potential conflict of interest.
